# Prevalence of Erectile Dysfunction in Chronic Hemodialysis Patients

**DOI:** 10.1192/j.eurpsy.2026.12054

**Published:** 2026-07-31

**Authors:** H. Hsine, S. Kolsi, A. Bouaziz, M. El Behi, W. Abbes

**Affiliations:** 1 Psychiatry, La Metairie Clinic, Nyon, Switzerland; 2 Psychiatry, University Hospital of Gabès, Gabes, Tunisia

## Abstract

**Introduction:**

Erectile dysfunction (ED) is a frequent but still insufficiently explored complication in patients with end-stage chronic kidney disease undergoing hemodialysis. It represents a relevant marker of overall health in these patients, closely linked to vascular, metabolic, and psychological disorders associated with renal disease. Despite its major impact on quality of life and marital relationships, ED remains rarely screened for and poorly managed in nephrology practice.

**Objectives:**

To determine the prevalence of ED in chronic hemodialysis patients and the associated factors.

**Methods:**

We conducted a descriptive and analytical cross-sectional study in the form of a survey among 50 patients with chronic renal failure treated with periodic chronic hemodialysis in the nephrology department of Gabes Regional Hospital. The study was carried out from February 1, 2025, to May 31, 2025. We used a pre-established form to collect sociodemographic and clinical data, and the Arabic version of the International Index of Erectile Function (IIEF-5) to screen for ED.

**Results:**

The mean age was 49.3 ± 16.18 years [19–78]. In our sample, 64% of patients were married. Their socioeconomic level was low in 46% of cases. Before initiation of hemodialysis, 86% of patients were professionally active. However, after starting dialysis, half of the patients became unemployed (56%). Hypertension was the most frequent comorbidity (70.7%). Nephropathy was of diabetic origin in 40% of cases. The mean duration of hemodialysis was 58 ± 82 months [3 months–25 years]. The mean IIEF-5 score was 12.94 ± 5.75. The prevalence of ED was 86%, moderate in 28% of cases, and severe in 40% of hemodialyzed men. We did not find statistically significant correlations between the IIEF score and age, somatic comorbidities (diabetes, hypertension, dyslipidemia), or dialysis duration.

**Conclusions:**

The high prevalence of ED further illustrates the particular severity of chronic kidney disease. It should be considered among the key determinants of quality of life in hemodialysis patients. Systematic screening for ED and a multidisciplinary management approach are essential.

**Disclosure of Interest:**

None Declared

